# Analysis of Non-Small Bowel Lesions Detected by Capsule Endoscopy in Patients with Potential Small Bowel Bleeding

**DOI:** 10.1155/2016/9063293

**Published:** 2016-03-22

**Authors:** Fatma Ebru Akin, Oyku Tayfur Yurekli, Aylin Demirezer Bolat, Mustafa Tahtacı, Huseyin Koseoglu, Eyup Selvi, Naciye Semnur Buyukasik, Osman Ersoy

**Affiliations:** ^1^Ankara Ataturk Research and Teaching Hospital Gastroenterology Department, 06800 Ankara, Turkey; ^2^Yıldırım Beyazıt University Faculty of Medicine, Ankara Ataturk Research and Teaching Hospital Gastroenterology Department, 06800 Ankara, Turkey

## Abstract

Gastrointestinal (GI) bleeding cases in whom source cannot be identified after conventional upper and lower GI endoscopy are defined as potential small bowel bleeding. We aimed to search for lesions in the reach of conventional endoscopy in patients to whom video capsule endoscopy (VCE) had been applied for potential small bowel bleeding. 114 patients who had VCE evaluation for potential small bowel bleeding between January 2009 and August 2015 were retrospectively evaluated. Mean age of the patients was 55 ± 17 years. Female/male ratio is 39/75. In 58 patients (50.9%) bleeding lesion could be determined. Among these 58 patients 8 patients' lesions were in the reach of conventional endoscopes. Overall these 8 patients comprised 7% of patients in whom VCE was performed for potential small bowel bleeding. Among these 8 patients 5 had colonic lesions (4 angiodysplasia, 1 ulcerated polypoid cecal lesion), 2 had gastric lesions (1 GAVE, 1 anastomotic bleeding), and 1 patient had a bleeding lesion in the duodenal bulbus. Although capsule endoscopy is usually performed for potential small bowel bleeding gastroenterologists should always keep in mind that these patients may be suffering from bleeding from non-small bowel segments and should carefully review images captured from non-small bowel areas.

## 1. Introduction

Bleeding from the small intestine accounts for about 5–10% of all cases presenting with gastrointestinal (GI) bleeding. With recent developments of the small bowel imaging techniques like video capsule endoscopy (VCE), deep enteroscopy, and radiographic imaging, the source of bleeding in the small bowel can now be identified in the majority of these patients. The term “potential small intestinal bleeding” is proposed for patients with GI bleeding in whom normal upper and lower GI tract examinations are normal and VCE is considered. Overt small bowel bleeding refers to patients presenting with either melena or hematochezia with a bleeding source identified in the small intestine. Occult small bowel bleeding refers to patients presenting with iron deficiency anemia with or without guaiac-positive stools who are found to have a small bowel source of bleeding [1]. VCE has been recommended as a first-line procedure by recent guidelines for small bowel evaluation for GI bleeding after upper and lower GI sources have been excluded [1–3].

With VCE noninvasive evaluation of the small bowel has become possible, with a diagnostic yield of 38–83% in patients with suspected small bowel bleeding [4]. Furthermore, it has been shown to have superior diagnostic yield compared to small bowel follow-through [5, 6], push enteroscopy [6, 7], and computed tomography enteroclysis [8] and similar to double-balloon enteroscopy in detecting bleeding small intestinal lesions [9].

Small bowel lesions were overlooked during conventional endoscopy, either because of intermittent bleeding nature of the lesions or because some lesions are really missed. In fact there are few studies showing bleeding lesions within the reach of conventional upper endoscopy/colonoscopy that have been obviously missed during the initial examination [10–13].

The aim of our study is to determine the incidence of lesions missed by the preceding conventional upper endoscopy or colonoscopy.

## 2. Materials and Methods

We reviewed prospectively collected databases of patients referred to the Digestive Endoscopy Unit of the Atatürk Education and Research Hospital in Ankara to undergo a VCE analysis between January 2009 and September 2015. 117 patients were referred to our tertiary referral center for potential small bowel bleeding. All patients had undergone at least one upper gastrointestinal endoscopy and colonoscopy before the VCE examination, which had failed to establish a bleeding source. Indeed 65 of the 117 patients had undergone an additional endoscopy or colonoscopy (55% of the study group). 89 patients were evaluated at another tertiary referral center (mainly university hospitals) and referred to our center and 28 patients were endoscopically evaluated at our center. Data about patient demographics, indications for the procedure, procedural data, and findings of the procedure were recorded. Non-small bowel lesions were defined as bleeding lesions proximal to the papilla of Vater or distal to the ileocecal valve. Small bowel lesions were defined as bleeding lesions located between papilla of Vater and ileocecal valve. Bleeding lesions were defined as lesions that absolutely or likely explain the cause of bleeding or anemia.

VCE was performed with the PillCam® capsule endoscopy system (Given Imaging, Yokneam, Israel). Until December 2014 VCE were performed by PillCam SB2. After that date due to renewal of the equipment VCE were performed by PillCam SB3. The patients were given standardized information before the procedure, and informed consent was obtained. The patients were put on low fiber diet 1 day before VCE procedure. Patients fasted for 10 hours before capsule ingestion. Bowel preparation consisted of 4 liters of polyethylene glycol preparation (Golytely®; Avicenna) or 250 mL sennoside A + B calcium (XM-Diet solution®; Yenişehir Lab.). The capsule was ingested at about 8:30 a.m. the next morning. At the end of the recording period, data recorder was removed and images were downloaded to the computer. The recordings of VCE were reviewed by two experienced gastroenterologists (FEA, OE). The most relevant findings obtained from VCE were documented and categorized according to standard terminology [14] as angioectasia(s), ulcer(s), active bleeding of unknown origin, erosion(s), polyp(s)/tumor(s), incidental abnormality of esophagus, stomach, and colon, and no abnormality.

All statistics were performed using SPSS 22 for Windows (SPSS Inc., Chicago, IL, USA).

## 3. Results

Capsule endoscopy was performed in 117 patients with potential small bowel bleeding. Three patients were excluded from the final analyses because of the capsule retention in the stomach. In one of these 3 patients capsule was trapped in gastric bezoar. The other patient had a history of gastric resection so capsule could not reach the small intestine. In the final patient reason of gastric retention could not be determined. Finally 114 patients were included in the study. Female/male ratio was 39/75 (34.2%/65.8%) and the mean age of the patients was 55,8 ± 17 years (range 18–88 years). VCE was performed for occult bleeding in 36 patients (31,6%) and for overt bleeding in the rest of 78 (%68,4) patients. In 58 of 114 (50.9%) patients definite or likely cause of the bleeding could be identified (Table 1). Among these 58 patients 8 patients' lesions were identified as non-small bowel within the reach of conventional endoscopy. All of these 8 patients were referred to VCE from another tertiary referral center (8/86, 9.3%). In none of the 28 patients who were referred to VCE from our center a non-small bowel lesion was present causing bleeding. 8 patients with non-small bowel lesions comprised 7% of all VCE procedures performed for potential small bowel bleeding. Five of these lesions were in the cecum, 2 were in the stomach, and 1 was in the duodenum (Table 2). The most common lesion in these non-small bowel sources of bleeding was angiodysplasia (5/8). Four of these 8 patients were actively bleeding at the time of VCE procedure. Detailed clinical information about these patients with non-small bowel source of bleeding is given below.

**Figure 1:**
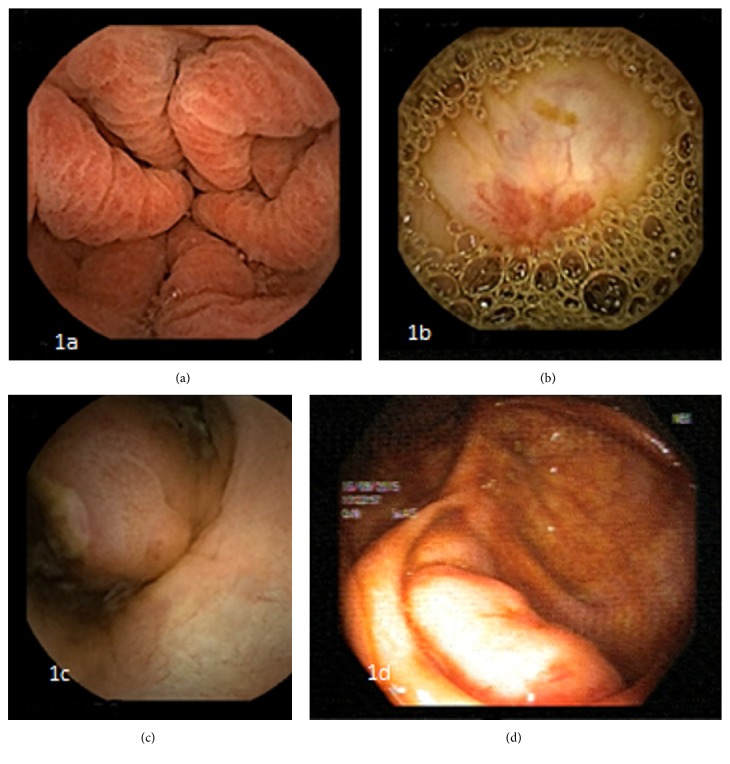
(a) CE and GAVE, (b) CE and angiodysplasia, (c) CE and ulcerated polypoid lesion, and (d) colonoscopy and ulcerated polypoid lesion. CE: capsule endoscopy; GAVE: gastric antral vascular ectasia.

In a patient with occult small bowel bleeding VCE revealed gastric antral vascular ectasia (GAVE) (Figure 1(a)). Previous upper endoscopy was reported as antral gastritis. Endoscopic treatment sessions with Argon Plasma Coagulation (APC) were performed.

In a patient with a history of Billroth II operation for peptic ulcer disease VCE detected active bleeding and ulcer on the gastric anastomosis. Upper endoscopy performed after VCE showed a hyperemic and fragile anastomosis line. Proton Pomp Inhibitor therapy was started.

One patient had active bleeding distal to the duodenal bulb during VCE. This patient had previously undergone upper GI endoscopy during active bleeding and was reported to have normal endoscopy findings. Lesion could not be identified with VCE because of active bleeding. But a repeat endoscopy after VCE revealed an angiodysplasia at this region and thermocoagulation was applied.

Three patients who applied for long lasting iron deficiency anemia had angiodysplasia (Figure 1(b)) in the colon and 1 patient had active bleeding during VCE. The colonoscopy performed after VCE showed colonic angiodysplasia in the patient with active bleeding from the colon. In none of these patients were previous colonoscopies able to define these lesions. Thermocoagulation was applied in 3 of these patients.

The patient who was found to have an ulcer in the cecum had applied for anemia and abdominal pain and colonoscopy findings before VCE were found to be normal. VCE revealed an ulcer on a protrusion in the cecum (Figure 1(c)). Colonoscopy performed after VCE showed a protrusion to the lumen in the cecum and an ulcer on top of it (Figure 1(d)). Biopsy taken from that area revealed chronic active colitis but the patient was lost to follow-up.

## 4. Discussion

It has been shown that 5–10% of GI system bleeding is small intestinal in origin. In patients with gastrointestinal bleeding in whom upper and lower GI endoscopies are normal small bowel should be considered as the site of bleeding. ACG recommends to classify these patients under the term of potential small bowel bleeding [1].

The clinical management of these patients with potential small bowel bleeding is controversial. VCE is a simple and noninvasive imaging technique for the examination of small bowel. Previous studies showed that VCE is able to show the source of bleeding in the small intestine in patients with potential small bowel bleeding [4, 15–20]. In patients with potential small bowel bleeding and in whom upper and lower GI system endoscopies cannot define the source of bleeding next step in the diagnostic algorithm is proposed to be VCE [1–3]. We were able to find definite or likely reason of bleeding in 50.9% of patients with potential small bowel bleeding who had undergone VCE. Interestingly in 7% of our patients bleeding lesion was in the reach of conventional upper or lower endoscopy. These patients had undergone at least one upper and lower endoscopy before the VCE procedure. The reasons of missing these lesions apparent on VCE with conventional endoscopy are unknown. One reason might be the small lesion size. Also some lesions being on unexpected sites might have caused missing of these lesions. Furthermore nonbleeding lesions during conventional endoscopy might have been noticed during active bleeding at the time of VCE examination. Half of our 8 patients with non-small intestinal cause of bleeding during VCE had vascular lesions. The patient who was diagnosed with GAVE was previously reported to have antral gastritis upon upper GI endoscopy. This patient's previous endoscopy was performed by an experienced endoscopist. This finding is in line with results of Kitiyakara and Selby [10]. They found 3 cases of GAVE in this study. These 3 patients' endoscopies were also performed by an experienced endoscopist and authors thought that this finding might be due to VCE's being performed under more physiological circumstances. Angiodysplasias detected at duodenal bulb or colon were not noticed upon previous conventional upper or lower GI endoscopy. Significant anemia or the patient being hypotensive during examination might have caused missing of these lesions. Lack of insufflation during VCE might have caused these lesions to seem more prominent. Lastly although these patients were referred from tertiary referral centers the level of expertise of the previous endoscopists was not exactly known. This may have caused the missing of these lesions. 4 patients had bleeding lesions in the cecum. Colonoscopists might have misidentified cecum in these patients and prematurely terminated the colonoscopy or these lesions might have been really missed because of being behind colonic haustrations. Furthermore incomplete bowel cleansing might have caused the missing of these lesions. Despite all of these potentially confounding factors current guidelines do not recommend second-look upper or lower GI endoscopies if the patient is not actively bleeding and first examination is optimal [1].

ACG recently proposed the term potential small bowel bleeding although the literature mainly comprised studies using the old terminological term obscure small bowel bleeding. There are a few studies describing non-small bowel reasons of obscure small bleeding upon VCE. Kitiyakara and Selby found 9 (6.4%) cases of non-small bowel bleeding among 140 patients who had undergone VCE for obscure small bowel bleeding. Results of this study show an apparent resemblance to our findings. 4 lesions were identified in stomach while 5 lesions were identified in the cecum [10].

In the study performed in 2011 by Vlachogiannakos et al. in 11 of 317 (3.5%) patients who had undergone VCE for obscure small bowel, the bleeding lesion was found outside the small bowel within the reach of conventional endoscopy. This study showed that repeating the conventional endoscopy for the second time in patients with obscure small bowel bleeding who have normal first upper and lower endoscopies was not cost effective [12]. Besides ACG clinical guideline recommends VCE as first-line procedure in potential small bowel bleeding patients after excluding upper and lower gastrointestinal lesions [1–3]. Second-look endoscopy and colonoscopy are recommended if indicated in patients with continuing active bleeding.

Another study found non-small bowel causes of bleeding in 54 out of 637 patients (8.5%) who had undergone VCE for obscure small bowel bleeding [13]. These studies show that in around 3.5–8.5% of patients with potential small bowel bleeding VCE was able to define the cause of bleeding within the reach of conventional endoscopy. We also found this ratio as 7% similar to previous reports.

## 5. Conclusion

In patients to whom small bowel VCE are applied for potential small bowel bleeding we can still find lesions responsible for the bleeding within the reach of conventional endoscopy. For this reason gastroenterologists evaluating VCE should thoroughly review also the gastric and colonic images in order to catch possible missed non-small bowel lesions. This approach will lead to planning of therapeutic endoscopic procedures which may improve patient outcomes.

## Figures and Tables

**Table 1:** Results of VCE performed for potential small bowel bleeding.

Lesions	*N* (114)
No abnormalities	56 (49.1%)
Angiodysplasia(s)	20 (17.5%)
Erosion(s)	5 (4.4%)
Ulcer(s)	17 (15%)
Polyp/tumor	7 (6.1%)
Active bleeding	7 (6.1%)
Other	2 (1.8%)

**Table 2:** Non-small bowel abnormalities in capsule endoscopy.

Patient number	Lesion	Age (years)	Gender	Clinical presentation of bleeding
1	GAVE	64	F	Occult
2	Active bleeding from gastric resection anastomosis site and ulcer	67	M	Overt
3	Active bleeding in the duodenal bulb	65	M	Overt
4	Angiodysplasia in the cecum	76	M	Overt
5	Angiodysplasia in the colon	50	M	Overt
6	Active bleeding from the cecum	70	F	Overt
7	Active bleeding from the cecum and angiodysplasia	80	F	Overt
8	Ulcer in the cecum	41	F	Occult
